# Lost Futures: The Human and Economic Cost of Suicide in Türkiye, 2012–2023

**DOI:** 10.3390/healthcare13222841

**Published:** 2025-11-08

**Authors:** Sevil Akbulut Zencirci, Emrah Atay, Muhammed Fatih Önsüz, Selma Metintaş

**Affiliations:** 1 Department of Public Health, Faculty of Medicine, Bilecik Şeyh Edebali University, 11100 Bilecik, Türkiye; 2 Independent Researcher, 26140 Eskişehir, Türkiye; emraha06@gmail.com; 3 Department of Public Health, Faculty of Medicine, Eskişehir Osmangazi University, 26040 Eskişehir, Türkiye; fatihonsuz@gmail.com (M.F.Ö.); selmametintas@hotmail.com (S.M.)

**Keywords:** suicide, years of life lost, economic burden

## Abstract

**Background/Objectives**: Suicide is a critical public health issue that leads to premature mortality among young people and requires substantial public health interventions. Demonstrating the importance of developing effective suicide prevention programs through health indicators can be valuable. This study aims to examine the change in suicide rates and Years of Life Lost (YLL) in Türkiye over the 2013–2023 period—a country that displays characteristics of both developed and developing nations—and to reveal the productivity losses using the human capital approach. **Methods**: The data for this descriptive study were obtained from the Turkish Statistical Institute between 2012 and 2023. YLL was computed by determining the difference between the age at which an individual died by suicide and their expected age of death for both males and females. Years of Potential Life Lost (YPLL) was estimated using the same method as YLL for individuals aged 15–64 (working age). The time trend of suicide and YLL rates was calculated using Annual Percentage Change and the Average Annual Percent Change values, based on a Poisson-based Generalized Linear Model and the Joinpoint regression method. Using the human capital approach, the economic cost of the labor force lost due to suicide was estimated. **Results**: The YLL per death was 33.57 years for males, 47.73 years for females, and 37.06 years overall. The age group with the highest YLL percentage is 20–24 among males (23.55%) and 15–19 among females (33.06%). An increase of 7.8% was observed among males from 2018 to 2021. The mean changes in the overall time trend were found to be significant in male suicides. Among females, there was a 5.3% decrease until 2017, followed by a 4.5% increase from 2017 onwards. Combined, male and female suicide rates have significantly increased since 2017. The financial loss associated with suicide over a 12-year period totaled USD 10,775,943,197 with an annual loss of USD 897,995,266. The premature mortality cost per death was estimated at USD 278,400.84 for men and USD 186,625.16 for women, while the premature mortality cost per YLL was USD 8292.23 for men and USD 3910.36 for women. **Conclusions**: Changes in the temporal trend of suicide may be associated with societal events. The study reveals that premature deaths due to suicide in Türkiye are a multidimensional public health problem that significantly affects not only individuals but also the overall productivity and economic structure of society.

## 1. Introduction

Suicide is a critical global health issue with serious consequences and requires public health intervention. According to the World Health Organization (WHO), annually, more than 720,000 individuals die by suicide, and many more attempt it [1]. Suicide can occur at any stage of life, and in 2021, it was reported as the third most common cause of death worldwide among people aged 15 to 29 [1].

Although suicide can be observed globally across all regions, low- and middle-income countries account for the majority of suicide deaths worldwide. The data from 2021 demonstrate that approximately three-quarters (73%) of suicide-related deaths occurred in these countries [1]. In developing countries, the limited capacity to respond to events such as pandemics, disasters, and economic crises increases individual vulnerability. The rise in suicide risk following these events highlights the seriousness of the issue [2,3,4,5]. Türkiye is a country located between the East and the West in terms of its socioeconomic, psychological, and cultural structure, and it possesses the features of both developed and developing countries [6]. In Türkiye, the COVID-19 pandemic and the economic crises of 2018 and 2023 have had significant societal impacts [7], underscoring the urgent need to evaluate suicide-related data and demonstrate the necessity of implementing effective prevention and management strategies.

Suicide is a multifaceted problem influenced by social, cultural, biological, psychological, and environmental factors that exist throughout the life span [1]. Consequently, the assessment of a multidimensional public health problem, such as suicide, in Türkiye necessitates consideration of socio-economic and cultural diversity. Indeed, it was reported that suicide rates in Türkiye may vary significantly over time based on age, gender, and methods [8]. There are substantial gaps in Türkiye’s national data concerning the causes of suicide [9]. While some causes of suicide, such as genetic factors, are unchangeable, others are modifiable and include issues like addiction, chronic illnesses, mental disorders, disability, and poverty [10]. Beyond individual-level determinants, suicide is also influenced by broader structural and environmental conditions. Urbanization, non-agricultural employment, and air pollution have all been identified as important factors affecting mental health and suicide risk [11,12,13]. The WHO explicitly states that suicide is preventable, and it therefore requires a multidimensional investigation of its causes [14]. Therefore, monitoring national and international trends in suicide can support the development of prevention programs, enable the evaluation of their outcomes, and allow for necessary adjustments over time. In this context, closely monitoring trends in suicide rates in Türkiye is essential, as the country displays characteristics of both developed and developing countries [6], and such data contribute to both national and international suicide epidemiology [14,15].

WHO characterizes suicide prevention as a “global imperative” urges nations to prioritize suicide prevention on the global public health agenda and to develop or strengthen comprehensive suicide prevention strategies employing a multisectoral public health approach [14]. The regular monitoring of suicide cases and the maintenance and analysis of up-to-date, reliable records are essential for the development of effective national suicide prevention strategies. International disparities in suicide rates and demographic characteristics underscore the necessity for countries to possess comprehensive and timely data specific to their own contexts [1,15]. While numerous nations have formulated and executed suicide action plans in accordance with the recommendations of WHO, Türkiye has not yet established a nationwide plan of this nature. This situation underscores the necessity of conducting research on the burden of suicide to inform policymakers. In the context of certain health programs, an exclusive emphasis on the number of deaths from preventable causes, such as suicide, may not adequately reflect the scope of the problem, hindering the development of effective control programs. Reporting of premature deaths and disease costs has been demonstrated to influence the prioritization of diseases in health policies [16,17].

Lucas argues that long-term economic growth, unlike classical and neoclassical growth models, is primarily driven by the accumulation of human capital. In this context, the sustainability of societal development depends heavily on the preservation and enhancement of human capital [18]. However, suicide—one of the leading causes of death among young people [1]—represents a significant threat to this development by causing the loss of human capital and, consequently, reducing productivity [19]. In the field of public health, some of the most important indicators used to measure human capital loss are Years of Life Lost (YLL) and Years of Potential Life Lost (YPLL) [19]. In this context, the Years of Life Lost (YLL) indicator, calculated based on age at death and case numbers, can be regarded as an effective and guiding measure for planning disease control programs and allocating resources to health services. Furthermore, Potential Years of Life Lost (YPLL) and economic costs associated with productivity loss, which are regarded as auxiliary indicators for assessing disease burden, are two additional significant measures employed to underscore the social and economic ramifications of premature deaths [20,21]. The phenomenon of suicide causes significant losses in a country by reducing both the quantity and quality of human capital, which is an important driving force of economic growth. Studies that address the phenomenon of suicide from such a perspective are limited in the literature [22,23].

This study has three primary objectives: (1) to examine temporal trends in suicide rates by gender and age groups from 2013 to 2023 using Joinpoint regression analysis; (2) to estimate the burden of suicide in terms of YLL and YPLL during the study period; and (3) to quantify the economic impact of suicide-related mortality through productivity loss calculations based on the human capital approach.

## 2. Materials and Methods

### 2.1. Data Sources

The data utilized in this study were obtained from suicide and population statistics published by the Turkish Statistical Institute (TurkStat) between 2012 and 2023. TurkStat is the official state agency that compiles and publishes suicide statistics in Türkiye [24].

Suicide statistics were gathered using the records of the General Directorate of Security and the General Command of Gendarmerie until 2012. Starting from that year, records obtained from TurkStat’s cause of death data, the Ministry of Justice’s Directorate General of Prisons and Detention Centers, and the General Staff Headquarters were also included, thereby expanding the scope of suicide statistics [9]. TurkStat makes suicide data available on its official website, along with certain variables it defines internally. Suicide data, the population of Türkiye by gender and age groups and life expectancy for that age group were obtained from TurkStat [9].

Productivity loss was calculated using several key economic indicators, including the annual average wage, the labor force participation rate, the discount rate, and the inflation rate. These metrics were obtained from the Organization for Economic Co-operation and Development (OECD) and The International Labor Organization (ILO) data for Türkiye [25,26]. Further measures pertaining to unemployment statistics and underutilization of the labor force were obtained from ILO [26].

TurkStat has been contacted regarding the use of the study data and has stated that the publicly available data published on the TurkStat website can be used without additional permission. Since an open database was used in the study, ethical approval is not required.

### 2.2. Statistical Analysis

#### 2.2.1. Calculation of Crude and Standardized Rates

In this study, suicide rates were calculated by gender and age group (under 15, 15–19, 20–24, 25–29, 30–34, 35–39, 40–44, 45–49, 50–54, 55–59, 60–64, 65–69, 70–74, 75 and older) using suicide data from TurkStat for the years 2012–2023. Gender and age-specific standardized suicide rates and 95% confidence intervals (CI) were computed using the standard population defined by WHO in 2002 [27]. Suicide rates were expressed as rates per 100,000 population.

#### 2.2.2. Joinpoint Regression

To analyze changes in suicide rates by gender and age group during the study period, the Joinpoint Regression Program (Version 5.4.0) was used. This program was initially employed to examine temporal changes in cancer mortality rates, and and has since been widely used in fields such as cardiology and psychiatry. This program is particularly useful for capturing temporal dynamics of an event [28]. The year ranges applied for each gender were not predetermined, as Joinpoint analysis determines both the number and timing of segments based on statistically significant trend changes over time. The most suitable point at which changes (increase or decrease) in suicide trends emerged was determined in the model. For each trend, the Annual Percentage Change (APC) in suicide rates and the corresponding 95% CI were calculated using a Poisson-based Generalized Linear Model [29]. The cells demonstrating multiple trends (increase or decrease) have multiple APC values. The Average Annual Percent Change (AAPC) value was also obtained throughout the time trend.

#### 2.2.3. Calculation of YLL and YPLL

YLL is a metric that reflects the difference between the age at death and the standard life expectancy [30,31]. The method used to estimate YPLL was similar to the estimation of YLL. In computing YPLL, all deaths above 65 years (the official retirement age in Türkiye) were excluded [32]. The working-age group for YPLL calculation was defined as 15–64 years in accordance with national and international demographic standards [33,34]. In this study, productivity-related YPLL was estimated using the human capital approach, which applies predetermined wage rates. This approach evaluates the economic loss due to premature deaths by considering an individual’s potential income and contribution to national productivity. These estimates reflect the projected loss of working time within a scenario analysis framework [35]. YLL and YPLL were subsequently represented as a percentage corresponding to each gender and age group for suicide. In other words, values for specific gender and age groups were calculated as percentages of the total YLL and YPLL attributed to all suicide cases.

YLL Murray formula [30,31]:

$$YLL=N\times [\frac{C\cdot e^{ra}}{(r+\beta {)}^{2}}\times (e^{-(r+\beta )a}\cdot [(r+\beta )L+1]-e^{-(r+\beta )(a+L)}\cdot [(r+\beta )L+1])]$$

where *N* is number of deaths, a is average age of the age group (Midpoint-Age), *L* is life expectancy remaining in that age group (Life-Expectancy), *C* is age weight constant (0.1658), *r* is discount rate (0.03), *β* is Age weighting parameter (0.04), *e* is Euler’s Constant.

YPLL formula:

$$\mathrm{YPLL}=\sum_{i=0}^{N}di\times (W_{U}-W_{L})$$

where *di* denotes the number of death at the midpoint of each age group; *W_U_* refers to the maximum working age (64 years), while *W_L_* indicates the minimum working age (ranging from 15 to 64 years) [36].

#### 2.2.4. Human Capital Approach

The human capital approach was used to estimate the economic cost of productivity loss due to suicide-related premature mortality in Türkiye. The human capital approach quantifies the societal worth of potential productivity and estimates the monetary impact of output losses based on market wage rates, stemming from disease-related morbidity and mortality [20].

Productivity-related YPLL was subsequently estimated through the human capital method, based on preset wage rates. This study employed the human capital method to estimate the economic cost of suicide-related premature mortality, taking into account individuals’ income and their contribution to national economic productivity. These estimates represent the loss of time due to work-related activities within the scope of the scenario analysis.

Productivity loss was computed using a formula that is the sum of the estimated value of earnings of individuals in the labor force (Y_ns_ × W_ns_ × P_ns_). The formula incorporates the annual average earning (Y), labor employment ratio (W) and the probability of survival (P) across each age group (n) and gender (s). The model was adjusted for changes in labor productivity (g), and lifetime earnings were discounted (i) to obtain their present value.

The human capital approach aims to estimate the economic value of an individual’s productive capacity. This method relies on discounting the potential future income stream of a person—such as wages earned throughout their working life—to its present value. However, in studies conducted at the national level, it is generally not possible to access individuals’ actual productive incomes. Therefore, certain assumptions are made; the minimum wage is used as a lower-bound indicator representing the lowest level of productivity in society. Some applications of the human capital method—particularly in estimating the economic burden of disease or premature mortality—have used the minimum wage as a proxy for individual productivity, especially when actual income data is unavailable [37]. The discount rate is a subjective parameter in economic evaluations and may vary depending on the context of a given country and methodological preferences. In methodological guidelines issued by international organizations, discount rates are generally considered within the 3–5% range, and a 3% rate is commonly used in public project appraisals [38,39,40]. In Türkiye, economic evaluation studies have similarly adopted 3% as a standard reference rate [41]. Accordingly, based on the economic data from the years analyzed in this study, a 3% discount rate and 8% labor productivity were adopted. Discount rate adjustment is applied to convert future cost values into their present equivalents [20,42]. This adjustment is required to reflect the impact of inflation (α). Inflation refers to the rise in overall prices of goods and services within an economy over a specific period, typically assessed using the Gross National Product metric [21,43].

Calculating Annual Salary Based on Annual Growth Rate:

$${cost}_{year}={cost}_{year-1}\times (1+\frac{{\mathrm{Annual}\;\mathrm{Growth}\;\mathrm{Rate}}_{year}}{100})$$

Annual Cost Calculation (Based on gender and total):

$${Cost\;\mathrm{of}\;\mathrm{Female}}_{year}={\mathrm{YLL}\;\mathrm{Female}}_{year}\times (\frac{{\mathrm{Female}\;\mathrm{Participation}\;\mathrm{of}\;\mathrm{Workforce}}_{year}}{100})\times {\mathrm{Salary}}_{year}$$

$${Cost\;\mathrm{of}\;\mathrm{Male}}_{year}={\mathrm{YLL}\;\mathrm{Male}}_{year}\times (\frac{{\mathrm{Male}\;\mathrm{Participation}\;\mathrm{of}\;\mathrm{Workforce}}_{year}}{100})\times {\mathrm{Salary}}_{year}$$

$${\mathrm{Total}\;Cost}_{year}={Cost\;\mathrm{of}\;\mathrm{Female}}_{year}+{Cost\;\mathrm{of}\;\mathrm{Male}}_{year}$$

Discounting to Present Value (with a 3% discount rate):

$${Cost\;\mathrm{with}\;\mathrm{Discount}}_{year}=\frac{{\mathrm{Total}\;Cost}_{year}}{(1+0.03{)}^{\left(\mathrm{Years}-\mathrm{First}\;\mathrm{Year}\right)}}$$

Although the inclusion of domestic production contribution in production loss calculations has been proposed in previous research [36], it was not included in this study due to the lack of reliable data on the subject in Türkiye. The costs were presented in US Dollars after the transformation using the average exchange rate of related year.

IBM SPSS Statistics (v22.0), Microsoft Excel for Microsoft 365 (v16.0.19328.20178) and R software (v4.4.3 for Windows) were used for analyses in the study.

## 3. Results

### 3.1. Crude and Standardized Suicidal Rate

The study revealed that during a 12-year period, the number of suicides among men was 31,741 (Crude Mortality Rate [CMR]: 6.50/100,000; Standardized Mortality Rate [SMR]: 6.19/100,000), while the number of suicides among women was 10,391 (2.14, 2.38, respectively), while the number of suicides among total was 42,132 (4.32, 4.20, respectively). The data indicate that 75.34% of suicide deaths were male, while 24.66% were female, resulting in a male-to-female ratio of 3.05 (Table 1). The mean age at death was 41.06 ± 18.11 for males, 37.45 ± 19.99 for females, and 40.17 ± 18.65 years overall. A statistically significant difference was observed in the age at death, with women a lower mean age at death observed among women (*p* < 0.001). The annual average number of suicides was 2645 ± 340.9 in men, 866 ± 92.4 in women, and 3511 ± 407.8 overall.

**Figure 1:**
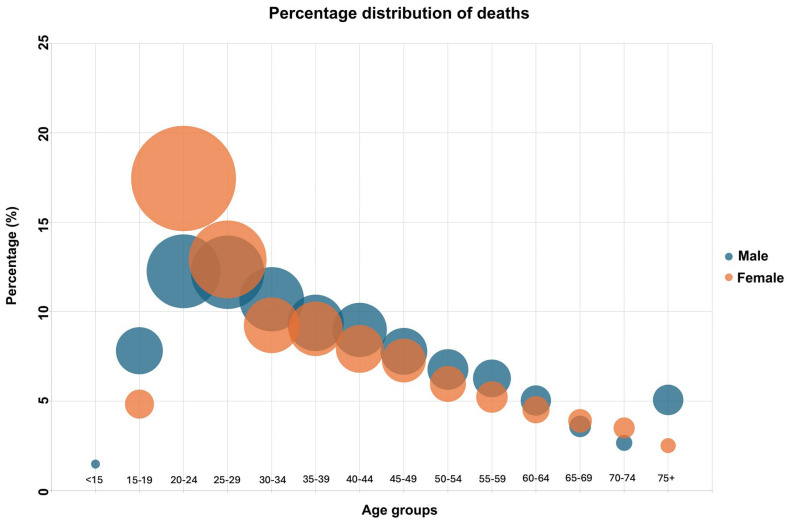
Percentage distribution of suicides by age group and gender.

Figure 1 shows the distribution of suicides by age group and gender. The highest death rates for both males and females are observed in the 20–24 age group. In this age group, the percentage of deaths out of total deaths was 12.26% for males and 17.45% for females. From this point onward, the death rates decrease progressively. An increase which breaks the descending trend is only observed in the percentage of the 75 and older age group among males.

### 3.2. YLL Due to Suicide

Suicide-related premature deaths over a 12-year period were 1,065,662.51 among men (Crude Rate: 218.31/100,000; Standardized Rate: 205.18/100,000), 495,918.90 (102.18/100,000; 108.07/100,000, respectively) among women and a total of 1,561,581.41 (160.42/100,000; 163.69/100,000, respectively) years over the same period. The average annual YLL for males, females, and total was 88,805.218, 41,326.57, and 130,131.78, respectively, while the YLL per death we 33.57, 47.73, and 37.06 years, respectively (Supplementary Table S1).

**Figure 2:**
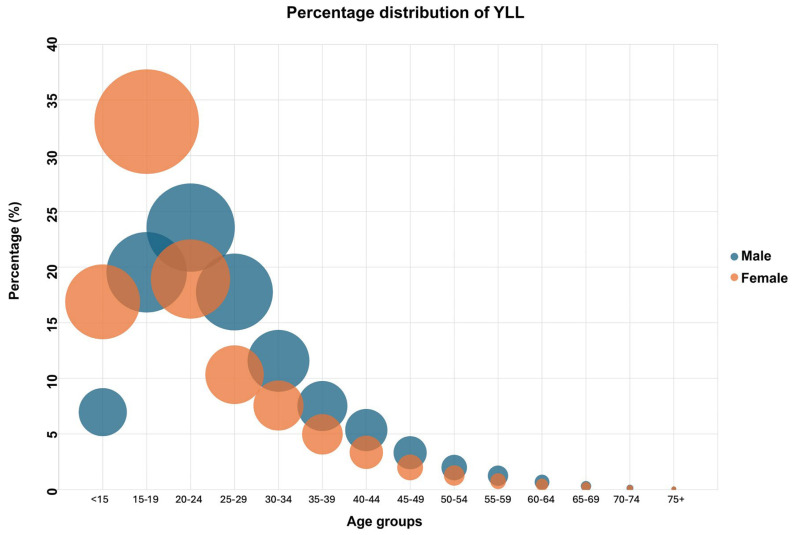
Percentage distribution of YLL due to suicide by age group and gender.

The percentage distribution of YLL due to suicide by age group and gender is shown in Figure 2. The age group with the highest YLL percentages among males is the 20–24 age group (23.55%). The age group with the highest YLL percentage among females is the 15–19 age group (33.06%). It is noticeable that YLL percentages gradually decrease after these age groups.

### 3.3. Time Trend of Suicides

Changes in suicide rate trends over time were analyzed using joinpoint regression. An increase of 7.8% was observed among males from 2018 to 2021. The mean changes in the overall time trend were found to be significant in male suicides. Among females, there was a 5.3% decrease until 2017, followed by a 4.5% increase from 2017 onwards. Taken together, male and female suicides have shown a significant increase since 2017.

**Figure 3:**
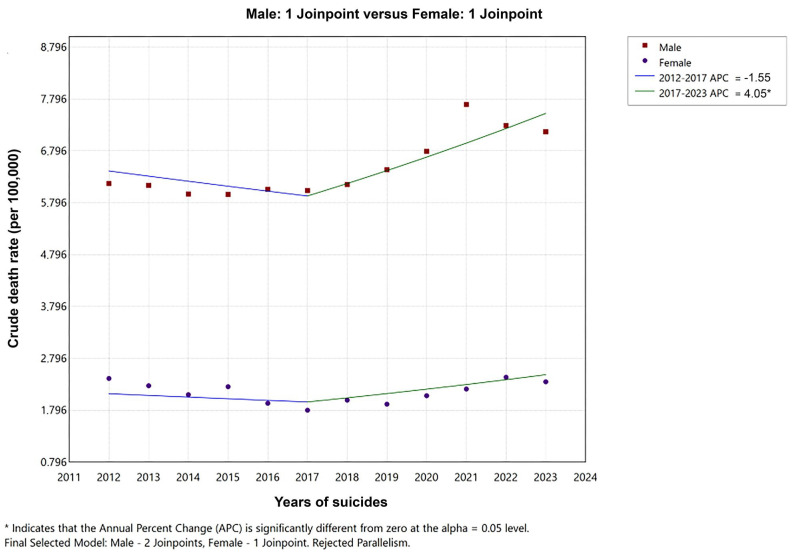
Joinpoint regression analysis: Trends in suicide rate (per 100,000) for males and females in Türkiye, from 2012–2023.

**Figure 4:**
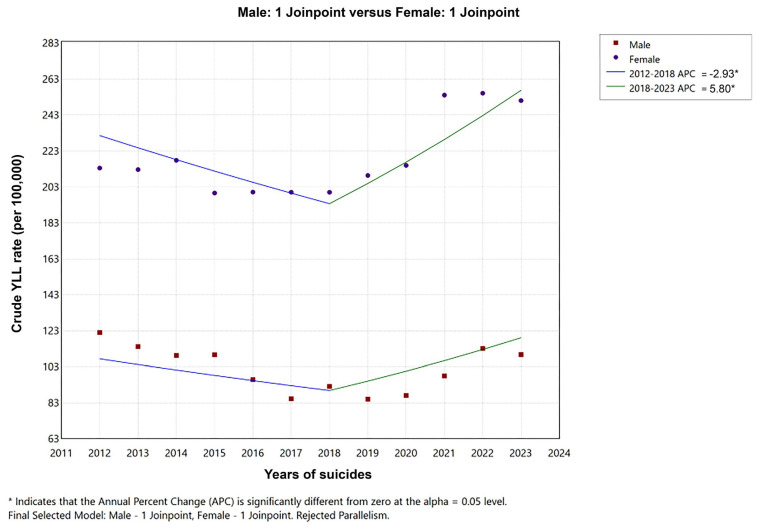
Joinpoint regression analysis: Trends in YLL rate (per 100,000) for males and females in Türkiye, from 2012–2023.

In males, a significant decrease in the YLL rate is observed in the 2012–2019 time trend, followed by a significant increase after 2019. In females, a notable upward trend in the YLL rate has been observed since 2018. When YLL rates due to suicide are combined across genders, a decreasing trend is evident from 2012 to 2018, followed by a significant increase after 2018. The mean changes in the overall time trend were found to be significant in female suicides (Table 2, Figure 3 and Figure 4).

### 3.4. YPLL Due to Suicide

The retirement age in Türkiye is 65, and the working age is considered to be 15–64, based on which the YPLL was calculated. The percentage distribution was found to be highest among men aged 25–49 and women aged 15–24 (Table 3).

### 3.5. Cost of Suicides Based on the Human Capital Method

The financial loss associated with suicide over a 12-year period totaled USD 10,775,943,197 with an annual loss of USD 897,995,266. Eighty-two percent of this amount was related to male suicides (Table 4).

Between 2012 and 2021, males experienced over 20% increases in YLL, YPLL, and financial losses, while during the 2017–2023 period females showed more modest increases ranging from 1.83% to 17.95% (Table 5).

## 4. Discussion

Suicide is a public health problem that requires priority intervention due to its serious social and economic consequences, especially in the productive age group. The findings of the study are important in revealing the extent of the impact on the young age group in an upper-middle-income country, as classified by the World Bank [44]. The study highlighted the necessity of examining suicide rates using time series analysis, while also evaluating them from a gender perspective and economically through cost estimation based on the human capital method.

According to WHO, in 2021 the suicide-related mortality rate in Türkiye was 2.7 per 100,000, which is lower than the world average (9.2 per 100,000) and is among the countries with low suicide mortality rates globally [45]. It has been suggested that many cultural, social and structural factors such as the deterrent effect of religious and social norms on suicide and the collectivist structure of the society in Türkiye may play a role in lower suicide-related mortality [46,47,48]. When evaluated by gender, although there are regional differences, suicide-related deaths are generally higher in males. Biological and social factors such as inadequate help-seeking behavior, inability to express emotions, work-related chronic stress, impulsive behaviors, alcohol and substance addiction, preference for lethal methods, hormonal effects, and gender-specific differences in drug metabolism have been put forward as the reasons why suicide is more common in men [49,50]. In a study conducted using WHO databases, it was reported that the male/female ratio of suicide-related deaths worldwide varied between 1.1 and 4 among WHO regions [51]. In our study, similar to this trend, male suicide deaths were found to be approximately three times higher than female suicide deaths. In addition, when the mean age at the time of death was analyzed, it was found that females had a lower age at death than males. In a study conducted in Switzerland, it was reported that women were more likely to attempt suicide at a younger age than men [52]. This finding of our study suggests that female suicide at a young age is a problem that should be prioritized in prevention efforts. Considering that suicides should be handled with a syndemic approach [53,54], it is important to identify other risky conditions accompanying suicides in young women. Risky situations such as adolescent pregnancies, female abuse and gender perspective [55,56,57] may have a role in increasing suicide in young women.

Suicide should be prioritized in health policies not only in terms of the number of deaths, but also in terms of the YLL at an early ages. Notably, the total YLL caused by suicide is higher than many infectious diseases, some chronic diseases and even some types of cancer [58]. In the study, total YLL due to suicide in Türkiye in the period 2012–2023 was calculated as 1,561,581 years, of which 68.25% were in men (1,065,662 years) and 31.75% in women (495,918 years). Although the number of suicide cases is higher in men, the average YLL per death is significantly higher in women (47.73 years vs. 33.57 years), indicating that suicides among women tend to occur at younger ages. These findings highlight the need for closer monitoring of the social and psychological risks faced by young women and strengthening gender-based preventive interventions.

In the study, an increase in suicide rates was observed among males starting in 2018 and among females starting in 2017. A significant rise in YLL due to suicide was also identified in the combined male and female population beginning in 2018. These findings suggest that suicide may be influenced by changing social, economic, and environmental conditions over time. One of the major events during this period of increase was the COVID-19 pandemic. The pandemic period was characterized by social isolation, uncertainty, intensified economic instability, and rising unemployment rates [59,60,61]. In the context of these conditions, factors such as the death of a family member, unemployment, job loss, and relationship problems may have increased the risk of suicide [62,63]. Indeed, several studies conducted in different countries have reported increases in suicide deaths and suicidal ideation during the COVID-19 pandemic [62,64,65]. After 2021, suicide rates among men began to decline again. Although the pandemic caused significant harm in many areas, the downward trend observed in 2021 may have served as a source of hope. This decrease may also have been influenced by factors such as the strengthening of social support mechanisms in the post-pandemic period, increased mental health awareness, and relatively improved access to healthcare services. Another major development during this period was the economic crises that occurred in Türkiye in 2018 and 2023. These crises had more severe consequences than previous periods of economic fluctuation, marked by rapid currency depreciation, high inflation, low growth, and increased personal debt [7]. Despite these conditions, individuals tended to sustain their consumption habits through borrowing and credit card use—a behavior associated with status quo bias, which may have intensified the individual-level impact of the crises [7]. These increasing economic pressures may have adversely affected mental well-being and contributed to an increased the risk of suicide. The findings indicate that extraordinary situations such as economic crises and pandemics may increase the risk of suicide. It is therefore important to develop policies that strengthen the psychological resilience of society and reinforce the supportive role of mental health and social services during such periods.

In this study, YPLL was calculated based on the working age group of 15–64 years in Türkiye. Of the total 1,040,325 years lost, 53.8% occurred in the 25–49 age group and 40.8% in the 15–24 age group. This distribution indicates that suicides are concentrated in the most productive and active periods of the society, thus posing a serious threat not only to life expectancy but also to national economic productivity. A gender breakdown reveals that the highest number of YPLL for men is in the 25–49 age group, while for women it is in the 15–24 age group. The high YPLL in young women highlights that this age group is psychosocially vulnerable and at increased risk for suicide. On the other hand, the heavy loss in the 25–49 age group among men suggests the impact of factors such as economic stress, occupational challenges, and family responsibilities on suicidal behavior during the productive age. Consistent with our findings, an Indian study reported the total economic burden of suicides for 2019 was calculated to be approximately 16.7 billion US dollars and it was found that the majority of this burden was caused by individuals in the 20–34 age group [22].

Based on the human capital method, the economic burden of premature deaths due to suicide in Türkiye was calculated as USD 10.78 billion over a 12-year period, with an average annual cost of approximately USD 900 million. This financial burden shows that suicide is an important public health challenge that affects not only the lives of individuals but also the productive potential of the national economy. Approximately 82% of the economic loss attributable to men and 18% to women. In the study, the average productivity loss per death was calculated as 278,400 dollars for men and 186,625 dollars for women, and the loss per life year was calculated as 8292 and 3910 dollars, respectively. In a study conducted in European Union countries with data from 2015, the loss of productivity due to suicide was estimated at €8.81 billion. It is reported that most of this loss is due to the death of men and corresponds to approximately 0.06% of the GDP of the European Union countries [19]. According to Lucas’s human capital-based growth model, deaths occurring at a young age reduce a nation’s long-term productive capacity [18]. In this context, the high YLL and YPLL values calculated in our study can be interpreted as a potential threat to Türkiye’s economic growth prospects. On the other hand, considering that each suicide may impact up to 135 people in the individual’s social circle [66], it is thought that the total economic burden may be much higher in reality. In addition to the measurable economic burden, suicide has profound socio-psychological effects on families, communities, and society at large, including grief, trauma, stigma, and long-term mental health consequences among those left behind [66]. The difference found between men and women may be related to the higher suicide rates in the productive age group among men, as well as the relatively low labor force participation rate of women in Türkiye [23]. The fact that the financial burden of housework was not taken into account in the study may also have led to a lower economic loss for women. Time series break analysis revealed that, between 2018 and 2021 in particular, YLL, YPLL, and economic loss indicators increased by more than 20% among men. A similar upward trend was observed for women, although not as pronounced as for men. The findings suggest that suicide is strongly influenced by periodic social and economic conditions, and therefore, suicide prevention policies should be considered as a priority investment in humanitarian and economic terms.

## 5. Limitations

The data used in this study are based on official mortality statistics of the TurkStat. WHO’s Global Health Estimates for international comparisons are prepared through modeling that includes standard methods such as reclassifying causes of death, estimating for missing years, and harmonizing with UN population projections in order to reduce differences in data quality across different countries. According to WHO, Türkiye is among the countries with a medium quality reporting system in terms of the accuracy of suicide data. Especially in sensitive issues such as suicide, there is a risk of underestimating the actual number of deaths due to data problems like misclassification (accidents or deaths recorded for unclear reasons, etc.) or underreporting [15]. Therefore, it should be taken into consideration that suicide rates and burdens calculated according to TurkStat data may not fully reflect the actual situation. In addition, considering that each suicide case affects many people directly or indirectly [66], suicide has social and economic effects not only on the individual but also on their surroundings; therefore, the total economic burden may be higher than the estimates produced using the human capital method. Another methodological limitation of the study is the use of the human capital approach in calculating the economic burden. This method does not fully reflect the actual productivity of individuals, as it often relies on minimum wage assumptions and does not account for variations in productivity based on education level, occupation, or sector. By assigning the same monetary value to all individuals, the method tends to obscure existing socioeconomic inequalities. Additionally, the underestimation of productivity loss, especially for women, is another limitation of the study, since the economic equivalent of unpaid domestic labor cannot be taken into account in the calculations. Furthermore, the study does not examine causal relationships between suicide and broader socio-economic indicators such as unemployment, income inequality, or education. As the primary aim was burden estimation, such analysis was beyond the scope of this paper.

## 6. Conclusions

The suicide surveillance system constitutes the cornerstone of suicide prevention programs. Producing high-quality and comprehensive data through surveillance systems is crucial for developing effective policies. Suicide is a complex phenomenon that cannot be reduced to a single cause. Therefore, suicide surveillance systems should be improved to uncover the chain of factors leading to suicide. Our findings clearly highlight the urgent need to strengthen suicide surveillance systems and to establish comprehensive suicide prevention programs both in our country and worldwide.

The study reveals that premature deaths due to suicide in Türkiye are a multidimensional public health problem that significantly affects not only individuals but also the overall productivity and economic structure of society. Suicide cases, which are particularly concentrated in the productive age group, result in significant reductions in both life expectancy and economic productivity. Many factors, such as gender- and age-specific differences, gender inequality, vulnerability of the young population, economic instability, and inadequate social support mechanisms, may contribute to suicidal behavior. Recent socioeconomic fluctuations and the COVID-19 pandemic have further increased these losses. In this context, suicide prevention interventions should not only focus on mental health services but also on eliminating inequalities in the social structure and reducing structural risk factors. The high economic cost of suicide deaths at an early age demonstrates that suicide prevention policies should be considered a priority investment in economic as well as humanitarian terms. Future research could address these associations using multivariate or causal methods based on disaggregated data.

## Figures and Tables

**Table 1:** Number of suicides by gender and crude and standardized rates per 100,000 population, Türkiye, 2012–2023.

Year	Male			Female			Total		
	Number	Crude Rate	Std. Rate *	Number	Crude Rate	Std. Rate *	Number	Crude Rate	Std. Rate *
2012	2341	6.17	6.19	907	2.41	2.38	3248	4.29	4.25
2013	2359	6.13	6.12	866	2.27	2.23	3225	4.21	4.14
2014	2325	5.96	5.91	811	2.09	2.07	3136	4.04	3.96
2015	2353	5.96	5.88	882	2.25	2.20	3235	4.11	4.02
2016	2424	6.05	5.96	767	1.93	1.90	3191	4.00	3.90
2017	2445	6.03	5.90	723	1.80	1.76	3168	3.92	3.81
2018	2529	6.15	6.00	813	1.99	1.96	3342	4.08	3.95
2019	2684	6.43	6.25	792	1.91	1.88	3476	4.18	4.05
2020	2845	6.79	6.58	865	2.07	2.01	3710	4.44	4.27
2021	3263	7.69	7.42	931	2.20	2.06	4194	4.95	4.77
2022	3111	7.29	7.05	1035	2.43	2.41	4146	4.86	4.72
2023	3062	7.17	6.92	999	2.34	2.32	4061	4.76	4.61
Total	31,741	6.50	6.19	10,391	2.14	2.38	42,132	4.32	4.20

* Standardized rate.

**Table 2:** Joinpoint regression analysis: trends in suicide rate (per 100,000) and YLL (per 100,000) for males and females in Türkiye from 2012–2023.

**Suicide Rate**									
**Male**				**Female**			**Combined**		
**Period**		**APC**	***p***	**Period**	**APC**	***p***	**Period**	**APC**	***p***
2012–2018		−0.3 (−1.4 to 0.5)	0.458	2012–2017	−5.3 * (−9.5 to −2.7)	0.010	2012–2017	−1.6 (−4.3 to 1.3)	0.267
2018–2021		7.8 * (5.9 to 9.3)	0.011	2017–2023	4.5 * (2.5 to 7.9)	0.0014	2017–2023	4.0 * (2.0–6.2)	0.001
2021–2023		−2.6 * (−5.0 to −0.3)	0.012						
AAPC	1.4 * (1.0 to 1.8)			0.0 (−1.0 to 0.9)			1.5 (−0.1 to 3.1)		
**YLL Rate**									
**Male**				**Female**			**Combined**		
**Period**		**APC**	***p***	**Period**	**APC**	***p***	**Period**	**APC**	***p***
2012–2019		−5.2 * (−7.5 to −2.9)	0.001	2012–2018	−1.3 (−3.8 to 1.4)	0.300	2012–2018	−2.9 * (−5.6 to −0.1)	0.040
2019–2023		8.2 * (2.2 to 14.6)	0.014	2018–2023	5.8 * (2.5 to 9.3)	0.004	2018–2023	5.8 * (2.1 to 9.6)	0.003
AAPC	−0.5 (−2.7 to 1.6)			1.9 * (0.2 to 3.7)			0.9 (−1.1 to 3.0)		

APC: Annual Percent Change, AAPC: Average Annual Percent Change, * *p* < 0.05.

**Table 3:** Potential years of life lost by gender and rates per 100,000 population in the 2012–2023 trend.

Age Group	Male			Female			Total		
	YPLL	YPLL per 100,000 Population	Distribution of Percentage	YPLL	YPLL per 100,000 Population	Distribution of Percentage	YPLL	YPLL per 100,000 Population	Distribution of Percentage
15–24	281,880.6	356.09	36.60	142,293.1	188.74	52.69	424,173.7	274.45	40.77
25–49	443,358.4	243.02	57.56	116,611.27	65.34	43.18	559,969.6	155.15	53.83
50–64	45,014.9	62.29	5.84	11,167.13	15.32	4.13	56,182.1	38.70	5.40
Total	770,253.9	230.71	100.00	270,071.5	82.65	100.00	1,040,325.4	157.47	100.00

**Table 4:** Financial loss values by gender using the human capital method related to suicide.

	Male	Female	Total
Total premature mortality cost (USD) (12 years period)	8,836,721,182	1,939,222,015	10,775,943,197
Total premature mortality cost (USD)/annual	736,393,432	161,601,835	897,995,266
Premature mortality cost per death (USD)	278,400.84	186,625.16	255,766.24
Premature mortality cost per YLL (USD)	8292.23	3910.36	6900.66
Premature mortality cost per YPLL (USD)	11,472.48	7180.40	10,358.24

**Table 5:** Changes in YLL, YPLL, and financial loss according to break periods determined by time trends.

Gender		YLL Annual Average Value (Year)	% Change	YPLL Annual Average Value (Year)	% Change	Cost Annual Average Value (USD)	% Change
**Segment**		**Male**					
1	2012–2018	80,889.40		57,160.01		653,758,685.3	
2	2018–2021	99,887.35	1–2 +23.49%	72,662.14	1–2 +22.36%	852,082,077.6	1–2 +25.56%
3	2021–2023 *	-		-		-	
**Segment**		**Female**					
1	2012–2017	41,014.29		21,633.46		150,358,046.50	
2	2017–2023	41,763.76	1–2 +1.83%	23,727.46	1–2 +9.68%	177,343,137.90	1–2 +17.95%

* Analysis could not be performed due to insufficient data.

## Data Availability

The data presented in this study were obtained from the publicly available resources of the Turkish Statistical Institute (TurkStat) at https://data.tuik.gov.tr/, accessed on 11 May 2023. The analyses and calculations are based on these publicly available data.

## Supplementary Materials

The following supporting information can be downloaded at https://www.mdpi.com/article/10.3390/healthcare13222841/s1. Table S1: Crude and standardized rates of years of life lost per 100,000 population by gender, 2012–2023 trend.
