# Supplementary material for: Lost Futures: The Human and Economic Cost of Suicide in Türkiye, 2012–2023
Source: Healthcare (Basel). 2025 Nov 8;13(22):2841. doi: 10.3390/healthcare13222841 (PMC12651982; doi:10.3390/healthcare13222841)
Supplement: Supplementary file 1 [file healthcare-13-02841-s001.zip › healthcare-3924287-supplementary.pdf]

Table S1. Crude and standardized rates of years of life lost per 100,000 population by gender, 2012–2023 trend

**Age**	2012	2013	2014	2015	2016	2017	2018	2019	2020	2021	2022	2023	**2012-2023**
							**Male**						
<15	85.77	63.13	95.43	58.16	67.88	20.65	54.22	64.70	37.73	56.05	66.79	56.38	63.65
15-19	507.25	579.55	578.29	448.12	469.39	168.67	468.40	512.91	520.20	561.48	605.72	583.31	525.89
20-24	602.81	602.03	605.34	603.26	496.65	489.64	565.44	592.19	644.25	752.55	757.58	793.26	634.17
25-29	366.01	388.71	382.17	416.35	433.03	604.38	451.89	457.76	534.38	663.54	672.08	649.50	489.10
30-34	277.54	256.66	268.64	264.26	302.08	427.26	294.40	314.83	332.93	411.12	425.25	416.33	318.27
35-39	184.11	187.98	182.14	185.38	179.86	251.92	198.99	205.85	213.51	261.73	255.30	267.30	210.38
40-44	144.22	145.01	133.75	141.16	175.95	225.40	146.04	149.42	154.13	197.07	178.11	191.19	160.60
45-49	113.15	112.72	115.78	113.45	117.85	176.46	128.22	103.92	118.20	124.94	98.56	106.83	112.96
50-54	95.14	86.71	74.02	69.56	76.75	112.62	66.11	85.45	72.51	78.55	68.55	57.34	75.29
55-59	63.04	58.83	52.47	69.39	55.75	93.96	50.12	59.93	57.38	52.45	43.84	40.84	54.30
60-64	39.10	35.17	36.69	34.26	31.90	64.28	43.58	35.64	41.06	37.86	33.45	26.73	36.21
65-69	23.92	24.51	21.46	25.18	20.72	57.01	23.36	19.03	21.29	27.11	20.10	17.73	22.06
70-74	17.06	18.08	12.42	12.27	16.47	33.69	16.82	13.27	16.48	14.89	13.84	14.37	14.99
75+	7.79	7.46	6.66	6.43	6.92	10.81	5.85	5.72	6.41	5.66	4.91	4.44	6.11
Total													
cYLL	211.91	211.11	216.30	198.20	198.79	0.16	198.71	207.95	213.87	253.02	254.38	250.49	218.31
sYLL	205.18	212.05	194.61	196.21	199.32	198.80	209.75	216.32	256.47	260.54	257.54	217.74	205.18
							**Female**						
<15	96.52	83.81	116.54	85.48	70.94	54.25	66.39	44.43	62.55	66.45	74.57	89.31	75.81
15-19	503.04	497.97	403.38	447.32	411.91	415.19	404.47	365.98	359.19	478.30	509.44	453.41	437.45
20-24	286.76	276.32	249.03	261.20	241.55	184.75	195.77	239.24	211.39	225.63	306.21	297.08	247.56
25-29	137.23	126.32	112.60	151.14	130.85	104.99	125.22	122.35	145.64	128.36	174.58	178.24	136.72
30-34	104.20	100.47	103.46	99.90	77.52	94.50	99.04	120.25	87.76	106.50	104.40	92.30	99.25
35-39	80.58	62.76	63.91	60.85	57.19	45.47	74.08	54.84	60.30	63.98	84.86	85.21	66.00
40-44	56.69	45.36	43.55	46.44	35.72	46.04	50.72	34.11	48.72	52.30	56.46	51.43	47.44
45-49	27.61	35.87	25.89	36.42	31.16	25.89	27.56	27.61	31.93	33.63	39.70	40.46	32.15
50-54	25.90	24.67	17.19	28.97	19.54	14.96	24.91	25.18	21.09	21.72	27.11	19.81	22.55
55-59	13.31	13.95	13.19	12.68	16.04	13.79	15.79	13.58	14.67	20.24	15.58	17.69	15.20
60-64	11.57	14.37	12.34	9.65	8.08	8.34	9.12	6.87	15.66	13.98	8.17	11.44	10.72
65-69	10.14	9.20	8.50	10.02	6.50	6.84	7.15	7.91	8.38	7.83	7.34	5.79	7.84
70-74	7.69	4.09	7.42	4.86	5.41	4.02	4.86	2.98	5.58	3.29	4.95	3.26	4.74
75+	2.60	2.37	1.98	2.42	1.75	2.10	1.53	1.82	2.02	1.67	1.39	1.51	1.90
Total													
cYLL	122.96	115.05	110.06	110.44	96.58	85.85	92.79	85.65	87.56	98.41	113.53	110.00	102.18
sYLL	124.33	117.01	113.34	113.84	100.31	89.97	98.21	91.53	94.62	107.70	124.60	121.81	108.07

cYLL: Crude rate of years of life lost; sYLL: Standardized rate of years of life lost
